# Rethinking Proteinuria During Vascular Endothelial Growth Factor-Axis Therapy: Toward a Biopsy-Informed Approach

**DOI:** 10.1016/j.ekir.2026.107045

**Published:** 2026-08-22

**Authors:** Yoshitaka Furuto, Shruti Gupta

**Affiliations:** 1 Department of Hypertension and Nephrology, NTT Medical Center Tokyo, Tokyo, Japan; 2 Division of Renal Medicine, Mass General Brigham, Boston, Massachusetts, USA; 3 Adult Survivorship Program, Department of Medical Oncology, Dana-Farber Cancer Institute, Boston, Massachusetts, USA; 4 Harvard Medical School, Boston, Massachusetts, USA

### Introduction

Vascular endothelial growth factor-axis inhibitors, including VEGF ligand-directed agents, such as bevacizumab and VEGF receptor (VEGFR)-targeted tyrosine kinase inhibitors (TKIs), are integral to the treatment of advanced solid tumors. Their kidney-related adverse effects, including hypertension, proteinuria, and kidney dysfunction, may alter dose intensity and treatment continuity. Consensus-based onconephrology practice appropriately emphasizes baseline blood pressure, kidney function, and quantitative proteinuria assessment, followed by close surveillance and supportive management during therapy.^1^ The more difficult question is what new or worsening proteinuria implies, particularly when anticancer benefit remains substantial and the kidney consequences of continuing therapy remain uncertain.

### From Binary Labels to a Spectrum of Coexisting Phenotypes

VEGF-axis nephrotoxicity is commonly classified into renal-limited thrombotic microangiopathy (TMA), reflecting glomerular endothelial injury, and podocytopathy or focal segmental glomerulosclerosis (FSGS), indicating podocyte injury. Foundational observations established VEGF inhibition as a cause of glomerular endothelial injury and TMA.^2^ An influential 8-year observational study subsequently reported an agent-weighted distinction, with TMA predominantly observed during VEGF ligand-directed therapy and minimal change disease/FSGS patterns during VEGF receptor-targeted TKI therapy.^3^ More recent biopsy-defined experience indicates that this distinction is clinically useful but incomplete. In a heterogeneous 21-patient series of biopsy-proven TKI-associated kidney injury that included VEGFR-targeted and non-VEGFR-targeted agents, TMA-like lesions predominated and FSGS occasionally coexisted.^4^ Lenvatinib-associated nephrotic syndrome with a biopsy-proven FSGS lesion likewise shows a clinically relevant podocyte-weighted pattern during VEGFR inhibition.^5^

Podocytes are a major local source of VEGF-A, which acts on adjacent glomerular endothelial cells and is required for endothelial homeostasis.^2^ Disruption of this paracrine dependency can destabilize the integrated glomerular filtration barrier, providing a biologically plausible basis for overlapping endothelial and podocyte lesions. Whether podocyte injury is direct, secondary to endothelial injury, or both remains uncertain.

A more useful clinical model is therefore a biopsy-informed spectrum of coexisting endothelial and podocyte injury phenotypes rather than a validated temporal continuum. This framework does not imply that nephrotoxic potential is equivalent across agents. VEGF-axis disruption may present predominantly as endothelial swelling, mesangiolysis, capillary wall remodeling, and TMA, or as severe proteinuria with podocyte injury and an FSGS-pattern lesion. In some patients, these components overlap. Agent type, duration of exposure, baseline kidney vulnerability, blood pressure trajectory, and timing of biopsy may influence which component dominates at recognition. Figure 1a summarizes this biopsy-informed interpretation.

**Figure 1:**
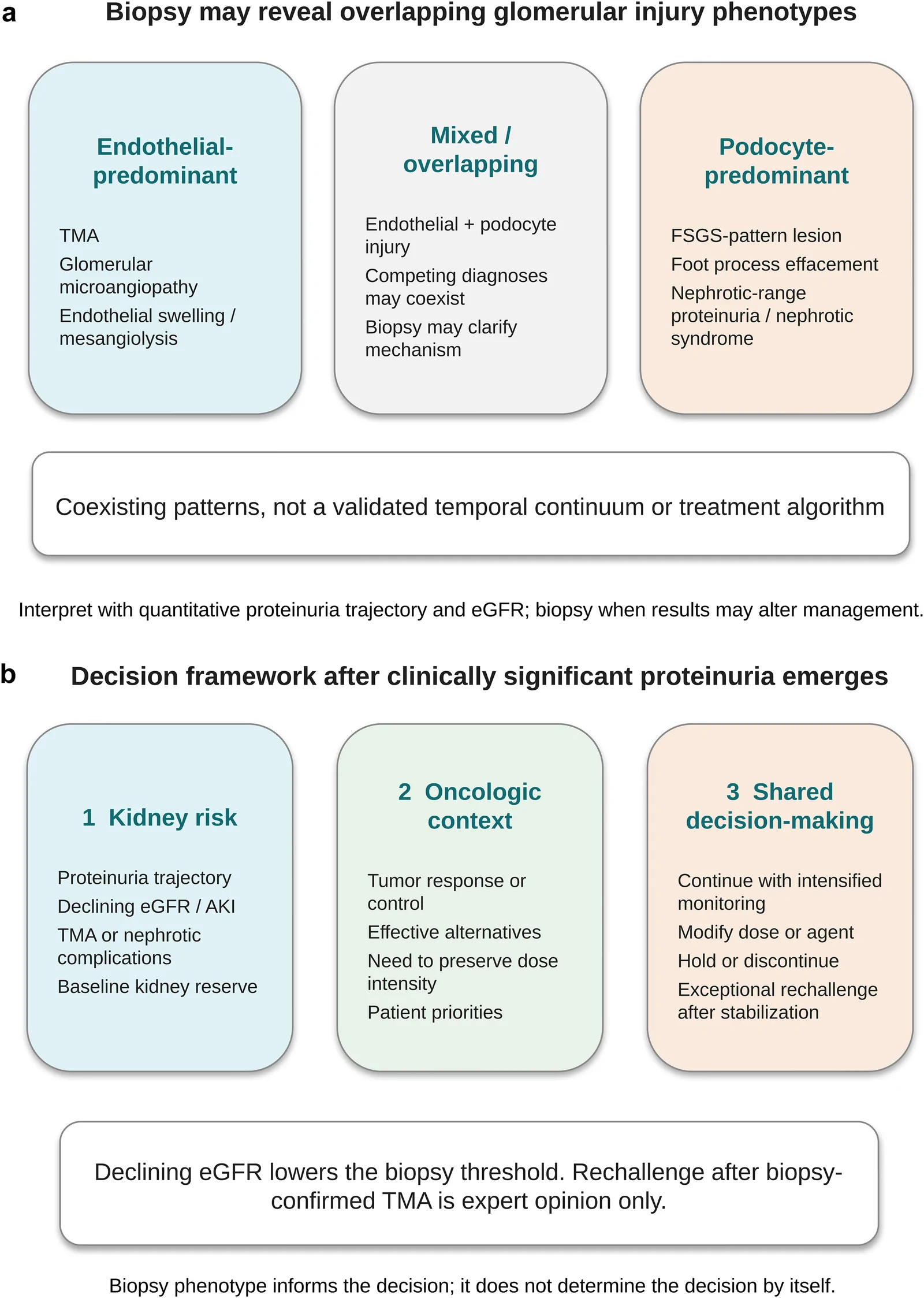
Biopsy-informed interpretation and decision-making during VEGF-axis therapy-associated proteinuria. (a) VEGF-axis inhibitor-associated glomerular injury may present as endothelial-predominant, mixed or overlapping, or podocyte-predominant phenotypes. These patterns may coexist and should not be interpreted as a validated temporal continuum or treatment algorithm. Clinically significant proteinuria should prompt reassessment, intensified surveillance, and consideration of kidney biopsy when the result could alter management. (b) When clinically significant proteinuria emerges, decisions should integrate kidney risk, anticancer benefit, treatment alternatives, and patient priorities. Proteinuria alone should not automatically mandate discontinuation of effective anticancer therapy. The figure was conceived by the authors; ChatGPT (OpenAI, GPT-5.6 Thinking) was used only to assist with visual simplification and layout. All scientific content and visual elements were reviewed and verified by the authors. eGFR, estimated glomerular filtration rate; FSGS, focal segmental glomerulosclerosis; TMA, thrombotic microangiopathy; VEGF, vascular endothelial growth factor.

### Proteinuria Is a Warning Signal, Not an Automatic Stop Rule

Throughout this Editorial, clinically significant proteinuria refers to persistent Common Terminology Criteria for Adverse Events version 5.0 grade 2 or higher proteinuria, a rapid quantitative increase, or nephrotic syndrome. In adults, Common Terminology Criteria for Adverse Events grade 2 corresponds to 2+ or 3+ dipstick proteinuria or urinary protein 1.0 to < 3.5 g/24 hours, and grade 3 to 4+ dipstick proteinuria or urinary protein ≥ 3.5 g/24 hours; when both are available, 24-hour urinary protein excretion takes precedence over dipstick grading.S1 Proteinuria signals possible glomerular injury but is not a complete surrogate for irreversible kidney failure. Persistent or rising proteinuria should not be dismissed as a benign class effect, even when blood pressure is controlled, because hypertension and proteinuria may reflect distinct mechanisms of VEGF-axis toxicity.

Product labeling provides safety rules rather than validated kidney prognostic thresholds. The bevacizumab prescribing information recommends further assessment with a 24-hour urine collection when dipstick proteinuria is 2+ or greater, withholding treatment for proteinuria ≥ 2 g/24 hours until it falls below 2 g/24 hours, and discontinuing treatment for nephrotic syndrome.S2 These label-based recommendations provide an operational safety framework, but their application should be individualized through multidisciplinary oncology-nephrology discussion, particularly when anticancer benefit is substantial. Their evidentiary basis for long-term kidney protection is limited, and they should not be extrapolated uncritically to all VEGFR-targeted TKIs or biopsy phenotypes. In addition, spot urine protein-to-creatinine ratio may support clinical reassessment but should not be treated as interchangeable with 24-hour protein excretion.S2 Proteinuria often improves after cessation, but resolution may be slow or incomplete; pooled bevacizumab data reported a median resolution time of 6.1 months, with nonresolution in 40% after a median 11.2 months of follow-up.S2

Clinical data remain conflicting. In a multicenter cohort, 44% of patients discontinued a VEGF signaling pathway inhibitor within 60 days after grade 2 or higher proteinuria, although reasons for discontinuation were not ascertained.^6^ In the ARISE study of 100 patients receiving atezolizumab plus bevacizumab, changes in the urine protein-to-creatinine ratio were not associated with changes in estimated glomerular filtration rate (eGFR), and the authors emphasized individualized risk-benefit assessment before bevacizumab interruption.^7^ In 70 patients receiving lenvatinib for radioiodine-refractory differentiated thyroid cancer, grade 3 versus grades 0 to 2 proteinuria was not associated with significant eGFR deterioration with dose or schedule adjustment.^8^ By contrast, a 2025 colorectal cancer cohort reported steeper subsequent eGFR decline among patients who developed proteinuria during bevacizumab treatment than among those who did not.^9^

These studies were not designed to evaluate outcomes by biopsy phenotype and are vulnerable to survival bias, immortal time bias, and confounding by indication. Findings from bevacizumab-based regimens may not apply to receptor-targeted TKIs or biopsy-proven microangiopathic injury. Taken together, the available evidence neither establishes the safety of continuing therapy despite heavy proteinuria nor shows that stopping therapy prevents kidney failure. To our knowledge, no study has directly compared long-term kidney and cancer outcomes among patients who continued versus interrupted treatment after clinically significant proteinuria emerged.

### When Biopsy Should Change Management

No validated proteinuria or eGFR threshold mandates biopsy. We would strongly consider biopsy when a 24-hour urine collection confirms persistent proteinuria near or above 2 g/24 hours. When a 24-hour collection is impractical, a markedly elevated or rapidly rising urine protein-to-creatinine ratio may support biopsy consideration, recognizing its imperfect concordance with 24-hour protein excretion. Biopsy should also be considered when proteinuria is nephrotic-range or rapidly increasing; when there is otherwise unexplained acute kidney injury or a sustained, clinically meaningful eGFR decline, for example, approximately 20% to 30% from baseline; or when active urine sediment, uncontrolled hypertension, suspected TMA, or complicated nephrotic syndrome is present. Declining eGFR should independently lower the biopsy threshold, even when proteinuria is only moderate, because it may indicate active endothelial injury or a competing process that cannot be resolved by proteinuria quantification alone.

The decision to perform kidney biopsy must be individualized. Thrombocytopenia, anticoagulation, anemia, uncontrolled hypertension, frailty, and cancer-related constraints may increase procedural risk or preclude biopsy. Nevertheless, a recent series of 318 image-guided nontarget kidney biopsies in selected patients with cancer reported a 97% diagnostic yield; adverse events occurred in 18%, most of which were low-grade, whereas grade 2 or higher events, including events requiring blood transfusion or embolization, occurred in 3.8%.S3 These data support feasibility in selected patients but do not eliminate the need for careful triage in patients treated with VEGF-axis inhibitors.

Biopsy should inform, not mechanically dictate, therapy. Active TMA features, including endothelial swelling, mesangiolysis, or thrombi, particularly when accompanied by ongoing eGFR decline, should generally favor holding, modifying, or switching the VEGF-axis therapy. Extensive chronic microvascular remodeling indicates limited remaining kidney reserve and should further lower tolerance for continued exposure. Podocyte-predominant injury is not necessarily benign. Collapsing features, diffuse foot process effacement with nephrotic syndrome, progressive eGFR decline, or thrombosis or severe edema should likewise favor interruption or treatment change. Conversely, podocyte-predominant injury without collapsing features, nephrotic complications, or progressive eGFR decline, accompanied by subnephrotic proteinuria and controlled blood pressure, may support cautious continuation when anticancer benefit is substantial. Biopsy may also identify an alternative immune-mediated process, especially during combination therapy with immune checkpoint inhibitors, for which treatment pathways differ.

The treatment decision must integrate biopsy findings with eGFR trajectory, symptoms, blood pressure, baseline kidney reserve, tumor response, effective alternatives, treatment duration, and patient priorities (Figure 1b). If therapy continues despite proteinuria, patients should undergo serial quantitative proteinuria and eGFR assessment, rigorous blood pressure management, renin-angiotensin system blockade when clinically appropriate, avoidance of additional nephrotoxins, and predefined triggers for biopsy, dose adjustment, treatment modification, or interruption.^1^ Sodium-glucose cotransporter-2 inhibitors may be used when otherwise indicated for diabetes mellitus, heart failure, or CKD, with attention to volume status and acute illness, but their efficacy specifically for VEGF-axis injury has not been established.

Rechallenge after biopsy-confirmed TMA is high-risk. Evidence is insufficient, and any rechallenge should be regarded as an exceptional, individualized expert-opinion decision rather than routine practice. If considered in exceptional circumstances, it should occur only after kidney stabilization, when anticancer benefit is substantial, effective alternatives are inadequate, microangiopathic risk is judged acceptable, and intensive monitoring with predefined stopping criteria is feasible. Heavy proteinuria alone should not invariably determine an anticancer treatment decision when its kidney prognostic meaning is uncertain, and the oncologic consequences of interruption may be immediate.

### What Evidence Is Now Needed

Prospective studies should compare continuation, treatment modification, and interruption after clinically significant proteinuria emerges, integrating proteinuria trajectories, eGFR slope, biopsy phenotype when available, kidney failure requiring kidney replacement therapy, anticancer dose intensity, tumor control, survival, and patient preferences. Trials should also test whether sodium-glucose cotransporter-2 inhibitors, mineralocorticoid receptor antagonists, glucagon-like peptide-1 receptor agonists, or endothelin receptor antagonists reduce VEGF-axis inhibitor-associated proteinuria and enable patients to remain on effective anticancer therapy. A pilot study has been registered to evaluate the efficacy and safety of sparsentan for VEGF signaling pathway inhibitor-associated proteinuria (NCT07224776). Until such evidence exists, biopsy-informed interpretation can identify serious kidney injury early, protect kidney reserve, and avoid reflexively sacrificing effective cancer therapy on the basis of proteinuria alone.

## Disclosure

YF serves as an unpaid councilor of the Japanese Society of Nephrology and reports no other competing interests. SG has served as a consultant for Mersana/DayOne Therapeutics, Alexion, BTG International, and MediBeacon. SG received research funding from BTG International, AstraZeneca, Johnson & Johnson, and Travere; research funding from NIDDK (K23DK125672, R03DK141708, R01DK148495, and R01DK144161); research funding from the MGB Department of Medicine; research support from the Chen Foundation; research support from the Claflin Foundation; and research funding from the American Heart Association.

## Supplementary Material

Supplementary File (PDF) **Supplementary References**.

## Footnotes

Supplementary File (PDF)

**Supplementary References.**
