# Supplementary material for: Rethinking Proteinuria During Vascular Endothelial Growth Factor-Axis Therapy: Toward a Biopsy-Informed Approach
Source: Kidney Int Rep. 2026 Aug 22;11(11):107045. doi: 10.1016/j.ekir.2026.107045 (PMC13594912; doi:10.1016/j.ekir.2026.107045)
Supplement: Supplementary file 1 [file mmc1.pdf]

Supplementary References

Rethinking Proteinuria During VEGF-Axis Therapy: Toward a Biopsy-Informed Approach

Manuscript ID: KIR-05-26-1183

S1. National Cancer Institute. Common Terminology Criteria for Adverse Events (CTCAE), Version 5.0. Bethesda, MD: National Cancer Institute; 2017.

S2. Genentech, Inc. Avastin (bevacizumab) [prescribing information]. South San Francisco, CA: Genentech, Inc.; 2022.

S3. Abdelsalam ME, Patel MN, Murray RD, et al. Percutaneous image-guided non-target renal biopsy in cancer patients: a tertiary cancer center experience. Curr Oncol. 2026;33:192. doi:10.3390/curroncol33040192.
